# Investigating Multi-Target Antiviral Compounds by Screening of Phytochemicals From Neem (*Azadirachta indica*) Against PRRSV: A Vetinformatics Approach

**DOI:** 10.3389/fvets.2022.854528

**Published:** 2022-06-16

**Authors:** Rajesh Kumar Pathak, Do-Young Kim, Byeonghwi Lim, Jun-Mo Kim

**Affiliations:** Department of Animal Science and Technology, Chung-Ang University, Anseong-si, South Korea

**Keywords:** *Azadirachta indica*, molecular docking, phytochemicals, PRRSV, pig, MD simulation, vetinformatics

## Abstract

Porcine reproductive and respiratory syndrome virus (PRRSV) is a global health problem for pigs. PRRSV is highly destructive and responsible for significant losses to the swine industry. Vaccines are available but incapable of providing adequate and long-term protection. As a result, effective and safe strategies are urgently needed to combat the virus. The scavenger receptor cysteine-rich domain 5 (SRCR5) in porcine CD163, non-structural protein 4 (Nsp4), and Nsp10 are known to play significant roles in PRRSV infection and disease development. Therefore, we targeted these proteins to identify multi-target antiviral compounds. To identify potent inhibitors, molecular docking of neem phytochemicals was conducted; three compounds [7-deacetyl-7-oxogedunin (CID:1886), Kulactone (CID:15560423), and Nimocin (CASID:104522-76-1)] were selected based on the lowest binding energy and multi-target inhibitory nature. The efficacy and safety of the selected compounds were revealed through the pharmacokinetics analysis and toxicity assessment. Moreover, 100 ns molecular dynamics (MD) simulation was performed to evaluate the stability and dynamic behavior of target proteins and their docked complexes with selected compounds. Besides, molecular mechanics Poisson–Boltzmann surface area method was used to estimate the binding free energy of each protein-ligand complex obtained from the MD simulations and validate the affinities of selected compounds to target proteins. Based on our analysis, we concluded that the identified multi-target compounds can be utilized as lead compounds for the development of natural drugs against PRRSV. If further validated in clinical studies, these compounds can be used individually or in combination against the virus.

## Introduction

Porcine reproductive and respiratory syndrome virus (PRRSV) causes a recalcitrant disease in pigs and responsible for major losses to the swine industry throughout the world (1, 2). Usually, the disease is further complicated by a secondary infection, leading to a high mortality rate. The PRRS virus was discovered in Europe and North America in the early 1990's. It is an encapsulated single-stranded positive-sense RNA virus from the genus *Porarterivirus* and family *Arteriviridae* (3, 4). There are currently four different species identified in this genus, PRRSV-1 and PRRSV-2 (30–45% sequence identity at nucleotide level), as well as lactate dehydrogenase elevating virus and rat arterivirus 1, which do not infect pigs (5, 6). Vaccination has been recognized as the primary method of disease control in the past years. The available vaccines, based on inactivated or modified live viruses, are incapable of providing adequate and long-term protection against PRRSV. As a result, effective and safe strategies are urgently needed to control PRRSV (1).

In the era of genomics and bioinformatics, it is easy to dissect intricate molecular mechanisms associated with host-pathogen interaction to identify new drug targets and candidates (7, 8). Additionally, bioinformatics is recognized as key disciplines in different areas of veterinary sciences. Consequently, the concept of vetinformatics has become a new approach for solving problems arising in the field of veterinary sciences by using computer science methodology (9). Furthermore, several developing countries have concentrated their efforts on developing human drugs, but only a few are working on the development of veterinary drugs (9). Therefore, the tremendous potential of vetinformatics for novel compound identification should be harnessed; it will directly help in livestock disease management, leading to an increase in productivity and sustainability.

Viruses are intracellular pathogens that replicate through a variety of host metabolic processes and encode proteins that facilitate their replication. Therefore, an efficient antiviral treatment must target virus-encoded proteins while leaving cellular metabolic processes unaffected (10). Unfortunately, many antiviral medicines that reduce virus replication also disrupt molecular processes in infected and non-infected cells. For many viruses, there is currently no known treatment. Plants are a rich source of antiviral compounds, and some have a wide range of antiviral potential with few or no side effects (10, 11). Various compounds previously derived from plants, e.g., isoscutellarein, 5,7-dimethoxyflavone, tetramethylluteolin, trimethylapigenin, 5-hydroxy-7-methoxyflavone, ginkgetin, quercetin 3-rhamnoside, celastrol, etc., have antiviral activity against influenza, H5N1, and SARS-CoV viruses (11–16). Furthermore, numerous FDA-approved antiviral medications such as famciclovir, sorivudine, ganciclovir, zidovudine, zalcitabine, didanosine, stavudine, and ivermectin are based on natural products (17, 18). Different medicinal plants are thought to be suppliers of powerful antiviral compounds. The neem tree (*Azadirachta indica*) belongs to the *Meliaceae* family and is a well-known medicinal plant in the Indian subcontinent. It is useful against a variety of ailments; its leaves, bark, fruit, flower, twig, gum, seed, and oil have medicinal properties (https://sites.google.com/site/neemdatabase1/importance/medicinal-and-agricultural-importance, accessed on 09/12/2021). In particular, it is used to treat skin problems, heat-rash, wounds, boils, jaundice, small pox, chicken pox, malaria, and other diseases (19). Besides, it offers highly effective, non-toxic, and environmentally friendly ways to control or eliminate insect pests and has potential applications in animal care and public health (20–22).

PRRSV infection is largely transmitted by porcine alveolar macrophages in the pig lung. A key receptor for PRRSV infection is CD163, a macrophage-specific membrane scavenger receptor (23–27). CD163 expression is required for PRRSV infection, as evidenced by knockout studies indicating that pigs lacking CD163 become PRRSV-resistant (28–30). Moreover, the scavenger receptor cysteine-rich domain 5 (SRCR5), one of the nine extracellular scavenger receptor cysteine-rich domains in CD163, is essential for PRRSV infection, and pigs with monocytes/macrophages expressing CD163 with deleted SRCR5 are completely immune to PRRSV infection (31, 32). Therefore, SRCR5 in porcine CD163 is one of the promising molecular targets for interrupting PRRSV infection; its crystal structure is also available in the public domain for further investigation (33). Additionally, previous studies revealed several other proteins and their involvement in PRRSV replication, growth, and pathogenesis, including non-structural proteins (Nsps) encoded by open reading frames (ORF1a and ORF1ab) in the PRRSV genome (5). This yielded at least fourteen functional Nsps (34). Out of these Nsps, functional and structural analysis found that Nsp4 and Nsp10 are essential in viral replication and pathogenesis, making them an important target for antiviral drug development. Furthermore, scientists determined their 3D structures through experimental techniques (35, 36).

**Figure 1:**
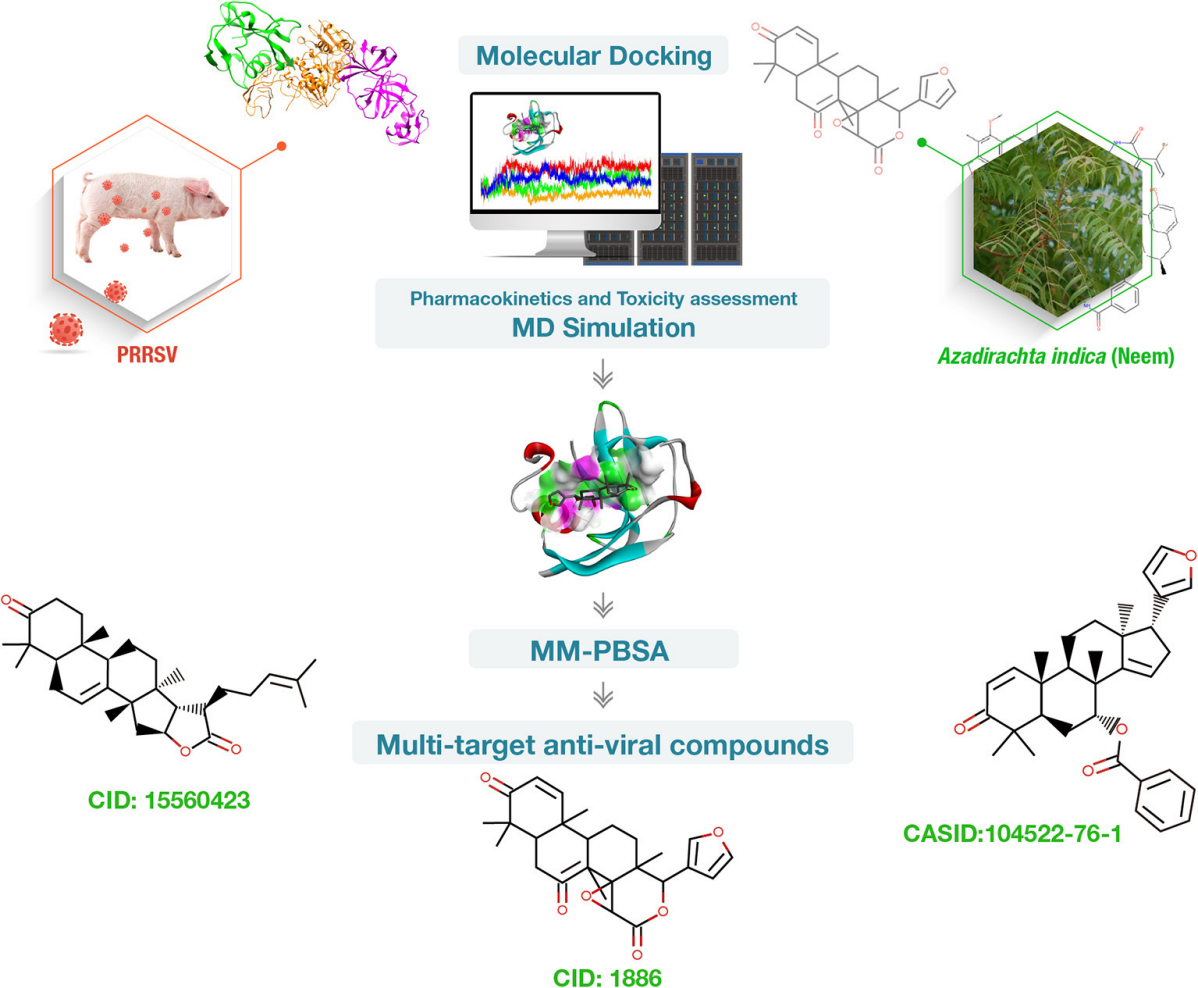
Summary of the work conducted to find phytochemical-based multi-target inhibitors of porcine CD163 scavenger receptor cysteine-rich domain 5 (CD163-SRCR5), non-structural protein 4 (Nsp4), and Nsp10 essential for porcine reproductive and respiratory syndrome virus (PRRSV) infection. Vetinformatics approaches were employed.

Therefore, phytochemicals present in neem can be utilized against PRRSV. The aim of our study is to use molecular docking, pharmacokinetics, toxicity assessment, molecular dynamics, and molecular mechanics Poisson–Boltzmann surface area (MM-PBSA) studies to investigate antiviral multi-target lead compounds using neem phytochemicals targeting porcine CD163 scavenger receptor cysteine-rich domain 5 (CD163-SRCR5), and PRRSV Nsp4 and Nsp10 (Figure 1).

## Materials and Methods

### Target Macromolecule Structure Retrieval and Receptor Grid Generation

The crystal structures of SRCR5 from porcine CD163 (PDB id: 5JFB), Nsp4 (PDB id: 5Y4L); and Nsp10 (PDB id: 6LKX) was retrieved from RCSB-Protein data bank (https://www.rcsb.org/) in pdb format and visualized by PyMOL (https://pymol.org/2/). AutoDock tools were used to prepare each retrieved structure for molecular docking by deletion of water molecules, addition of partial atomic charges (Kollman charge), and hydrogen atoms (37). The resultant structures were saved in pdbqt [Protein Data Bank (PDB), partial charge (Q), and atom type (T)] file format. The grid box size was generated to encompass all possible binding sites documented in literature for each target protein.

### Retrieval and Preparation of Neem Phytochemicals

A curated database Indian Medicinal Plants, Phytochemistry and Therapeutics (IMPPAT, https://cb.imsc.res.in/imppat/) was utilized in the present study. It holds 1,742 Indian medicinal plants and 9,596 phytochemicals along with other related information (38). The 3D structures of 70 neem phytochemicals were downloaded from IMPPAT database in pdb file format. Further, OpenBabel program (https://openbabel.org/wiki/Main_Page) was used to convert the file format from pdb to pqbqt to predict the binding free energies with selected target protein(s) and determine amino acid residues involved in protein-ligand interactions.

### Molecular Docking and Visualization

Molecular docking of 70 neem phytochemicals with CD163-SRCR5, and PRRSV Nsp4 and Nsp10 was carried out using AutoDock Vina (39). AutoDock Vina is an open-source molecular docking and virtual screening program that requires 3D structure of receptor and ligand molecules in pdbqt file format to predict their binding energy within receptor-ligand interaction studies. The docked protein-ligand complexes were generated by PyMOL (https://pymol.org/2/). Furthermore, Discovery Studio Visualizer was employed to visualize interacting amino acid residues and different bonding types formed during interactions (https://discover.3ds.com/discovery-studio-visualizer-download).

### Physicochemical Properties and Toxicity Studies

The physicochemical properties and toxicity analyses of predicted multi-target phytochemicals, i.e., CID:1886, CID:15560423, and CASID:104522-76-1, were performed to evaluate their drug-likeness in accordance with the Lipinski Rule of Five (40). Molecular weight, LogP, H-bond donor and acceptor, and topological polar surface area values were retrieved from IMPPAT (https://cb.imsc.res.in/imppat/) and PubChem (https://pubchem.ncbi.nlm.nih.gov/) databases, whereas mutagenicity, tumorigenicity, and irritation were predicted by OSIRIS Property Explorer (https://www.organic-chemistry.org/prog/peo/).

### Molecular Dynamics (MD) Simulation

The Gromacs (GROningen MAchine for Chemical Simulations, v2018.1) GPU-accelerated MD package was used to perform MD simulation studies (41, 42). A total of 12 systems were generated for MD simulations. Out of 12 systems, three estimated the dynamic behavior of target proteins CD163-SRCR5, Nsp4 and Nsp10, and the other nine estimated the dynamic behavior of the protein-ligand complexes. ProDRG was used to generate the ligand topology, whereas the GROMOS9653a6 force field was used to create the target protein topology (43–45). To reduce steric hindrance, all systems were subjected to the steepest energy minimization to achieve a peak force below 1,000 kJ mol^−1^ nm^−1^. To maintain the volume, temperature, and pressure, the systems were equilibrated, and position-restraint simulations were run under NVT and NPT conditions (46). Finally, a 100 ns MD simulation was conducted for all systems; the coordinates were stored at 2 fs intervals. The conformation stability, structural flexibility, structural compactness, protein–ligand contacts, and principal component analyses were conducted after a successful simulation using Gromacs utilities (https://www.gromacs.org/). Further, Xmgrace (https://plasma-gate.weizmann.ac.il/Grace) was utilized to plot the data and render the images.

### MM-PBSA Binding Free Energy Calculations

To support the previous findings, the binding free energy of each protein–ligand complex obtained from MD simulations was estimated quantitatively by the widely accepted MM-PBSA method (47). Snapshots of the last 5 ns of an MD trajectory were used to perform the MM-PBSA-based binding free energy calculation. The xtc, tpr, and index files generated during MD simulation were used. The van der Waals and electrostatic forces, polar solvation, solvent accessible surface area (SASA), and binding free energy were calculated using g_mmpbsa program (48).

## Results

### Screening of Neem Derived Phytochemicals Through Molecular Docking

Molecular docking can be used to investigate the best intermolecular framework formed between a macromolecule and a small molecule, such as a drug. It is a powerful computational approach and has a tremendous potential in identifying lead compounds for novel drug discovery. We used the molecular docking program AutoDock Vina to determine intermolecular interactions between selected target proteins and 70 neem phytochemicals. The phytochemical binding energy was predicted in the ranges of −2.3 to −6.8, −2.9 to −8.2, and −3.1 to −8.7 kcal/mol for CD163-SRCR5, Nsp4, and Nsp10, respectively (Supplementary Table 1). The top ten phytochemicals with the lowest binding energy [binding energy ranges: −6.8 to −6.0 (CD163-SRCR5), −8.2 to −7.3 (Nsp4), −8.7 to −7.9 (Nsp10) kcal/mol] were considered for further analysis to identify multi-target lead compounds.

Furthermore, favorable reactions have a negative free energy. Therefore, the lower the binding energy, the better the ligand-protein binding. The CID:1886, CID:11988279, and CASID:104522-76-1 showed the lowest binding affinities with CD163-SRCR5, Nsp4, and Nsp10, respectively. The CID:1886 and CASID:104522-76-1 had stronger interactions with the selected molecular targets, i.e., CD163-SRCR5, Nsp4, and Nsp10. However, CID:11988279 showed lowest energy with Nsp4 but higher energy with CD163-SRCR5 and Nsp10. Therefore, it could not be considered a multi-target compound. The top three out of top 10 screened multi-target compounds were selected based on the lowest binding energies with the selected molecular drug targets. Based on the result analysis, 7-deacetyl-7-oxogedunin (CID:1886), kulactone (CID:15560423), and nimocin (CASID:104522-76-1) were predicted as antiviral multi-target lead compounds against PRRSV, which inhibit CD163-SRCR5, Nsp4, and Nsp10. The top 10 screened phytochemicals, their binding energy with different target proteins, and amino acid residues involved in protein-ligand interactions are depicted in Table 1.

**Table 1:** List of the top ten screened neem phytochemicals, their binding free energies, and interacting amino acid residues of target proteins [porcine CD163 scavenger receptor cysteine-rich domain 5 (CD163-SRCR5), and porcine reproductive and respiratory syndrome virus (PRRSV) non-structural protein 4 (Nsp4) and Nsp10 of PRRSV].

**S.N**.	**Compound Name / ID**	**Binding energy (Kcal/mol)**	**CD163-SRCR5 interacting amino acid residues**	**Compound Name / ID**	**Binding energy (Kcal/mol)**	**Nsp4 interacting amino acid residues**	**Compound Name / ID**	**Binding energy (Kcal/mol)**	**Nsp10 interacting amino acid residues**
1.	**7-Deacetyl-7-oxogedunin/** **CID:1886**	**−6.8**	Ser504, Asp505, Phe506, **Ser507**, Ala510, Glu543, Phe544, Gln545, Cys546, Glu547, Pro562	Campest-4-en-3-one/ CID:11988279	−8.2	Phe3, Thr5, Ser9, Leu10, Asn11, Phe26, Val76, Pro78, Tyr92, Leu94, Val99, Pro101, Ile123	**Nimocin/ CASID:104522-76-1**	**−8.7**	Thr176, Asp179, Met180, Ala183, Asp225, Glu226, Gly152, Gly154, Lys155, Thr156, His157, Trp277, **Arg278**, Gly336, Ala337, **Thr338**
2.	Kulinone/ CID: 44567124	−6.8	Asp503, Ser504, Phe506, **Ser507**, Glu509, Ala510, Leu526, Leu527, Gly528, Phe544, Gln545, **Cys546**, Val572	24-Methylenecycloartan-3-one/ CID:14635659	−8.2	Phe3, Thr5, Ser9, Pro78, Tyr92, Leu94, Val99, Glu100, Pro101, Ile123, Thr124, Glu125, Ala126, Gly127	Melianin B/ CID:101650342	−8.5	Gly152, Gly154, Thr156, Gln175, **Asp179**, Arg182, Ala183, Trp277, **Arg278, Ser333**, Ser334, Gly336, Ala337, **Thr338, Arg365**
3.	**Kulactone/** **CID:15560423**	**−6.5**	Ile539, Trp540, Ala541, Glu542, Ala559, Pro560, Arg561, Asp563, **Gly564**, Thr565, Cys566	**7-Deacetyl-7-oxogedunin/ CID:1886**	**−7.7**	**His39**, Gly63, Asp64, Ser118, Thr134, Gly135, Ser136, Ile143, Thr145, Phe151	**Kulactone/** **CID:15560423**	**−8.5**	His34, Val71, Pro72, Tyr73, Lys74, Leu126, Pro127, Thr128, Pro172, Thr173, Leu212, Ala213, Tyr229, Cys230, **Asn231**, Asp234, Pro255, Val256
4.	**Nimocin/** **CASID:104522-76-1**	**−6.2**	His494, Gly537, Gln538, Ile539, Ala541, Glu542, Pro560, **Arg561**, Gly564, Thr565, His568	**Nimocin/** **CASID:104522-76-1**	**−7.5**	Ala38, His39, Val61, Lys62, Gly63, **Asp64**, Thr134, Ile143, Thr145, Ser148, Gly149, Phe151	Nimbolin A/ CID:101650373	−8.4	Gly149, Pro150, Pro151, Gly152, Gly154, Lys155, **Thr156**, Asp179, Arg182, Ala183, Glu226, Gln252, Arg278, Asp326, Gln335, Gly336, Ala337, Thr338, Arg365
5.	Meldenin/ CID:101289833	−6.2	Asp503, Ser504, Asp505, Phe506, **Ser507**, Ala510, Leu526, Glu543, Phe544, Gln545, Val572	Meldenin/ CID:101289833	−7.4	Thr5, Ser9, Leu10, **Asn11**, Phe26, Val76, Pro78, Tyr92, Leu94, Val99, Ile123, Gly127	**7-Deacetyl-7-oxogedunin /CID:1886**	**−8.2**	**Arg312**, Gly314, Asp326, Gly327, **His395**, Arg396, Asp397, Glu398, **Arg428**
6.	Nimbidinin/ CID:101306757	−6.2	Ser504, **Asp505**, Phe506, **Ser507, Leu508**, Glu509, Ala510, Glu543, Phe544, Gln545, **Cys546**, Glu547	Beta-Carotene/ CID:5280489	−7.4	Ala38, His39, Asn44, Val61, Gly63, Asp64, Thr134, Asn137, Ile143, Thr145, Phe151, Asn153, Val154, Ser180, His181	Isonimocinolide /CID:184310	−8.1	Tyr229, Leu253, His254, Pro255, Val256, Gly257, **Phe258, Asp259**, Asp332, Asn353, Gln355, Arg356
7.	Isoquercitin/ CID:10813969	−6.2	Ser504, Asp505, Phe506, Ser507, **Glu543**, **Gln545**, Cys546, Glu547, Pro562	Nimbaflavone/ CID:14492795	−7.3	Thr5, Lys7, Pro8, Ser9, Leu10, Asn11, Val76, Pro78, Tyr92, Val99, Pro101, Ile123, Thr124, **Glu125**, Ala126, Gly127	Beta-Amyrin/ CID_225689	−8.1	Gly152, Ala153, Thr156, His157, Asp179, Arg182, Ala183, Trp277, Arg278, Gly336, Ala337, Thr338
8.	Gedunin/ CID:114923	−6.2	Asp503, Ser504, Asp505, Phe506, Ser507, Leu526, Leu527, Gly528, Arg570, Val572	**Kulactone/** **CID:15560423**	**−7.3**	Ser19, Ala38, His39, Val61, Gly63, Asp64, Cys115, **Gly116**, Asp117, **Ser118**, Thr134, Gly135, Ser136, Ile143, Thr145, Phe151	MELIANONE/ CID_44575793	−8.1	Ala19, Pro36, Ile38, Gly42, His43, Pro44, Tyr73, Pro75, Arg77, **Thr78, Gln96**, Thr97, Ala121, Thr123
9.	7-Deacetyl-7-benzoylgedunin/ CID:52952112	−6.2	Asp503, Ser504, Asp505, Phe506, Ser507, Leu508, Leu526, Glu543, Phe544, **Gln545**, Cys546, Pro562	beta-Amyrin/ CID:225689	−7.3		Meldenin/ CID:101289833	−8	**Gly152**, Ala153, **Gly154**, Thr156, His157, Asp179, Met180, Ala183, Asp225, Glu226, Trp277, **Arg278**, Gly336, Thr338, Arg365
10.	Melianin B/ CID: 101650342	−6.0	**Arg1**, His494, Gln538, **Ile539**, Trp540, Ala541, Val558	7-Deacetyl-7-benzoylgedunin/ CID:52952112	−7.3	Ala38, His39, Val61, Gly63, Asp64, Thr134, Ile143, Thr145, Phe151	17-(3-Furyl)-4,4,8-trimethyl-3,16-dioxo-1,2:14,15-diepoxyandrostan-7-yl acetate /CHEMSPIDER:298060	−7.9	**Gly152**, Ala153, **Gly154**, Thr156, His157, Trp277, **Arg278, Thr338, Arg365**

*Compound names in bold represent the compounds predicted to be multi-target lead compounds, and amino acid residues in bold indicate the residues involved in hydrogen bonding interactions*.

### Top-Screened Multi-Target Phytochemical-Protein Complex Analysis and Visualization

**Figure 2:**
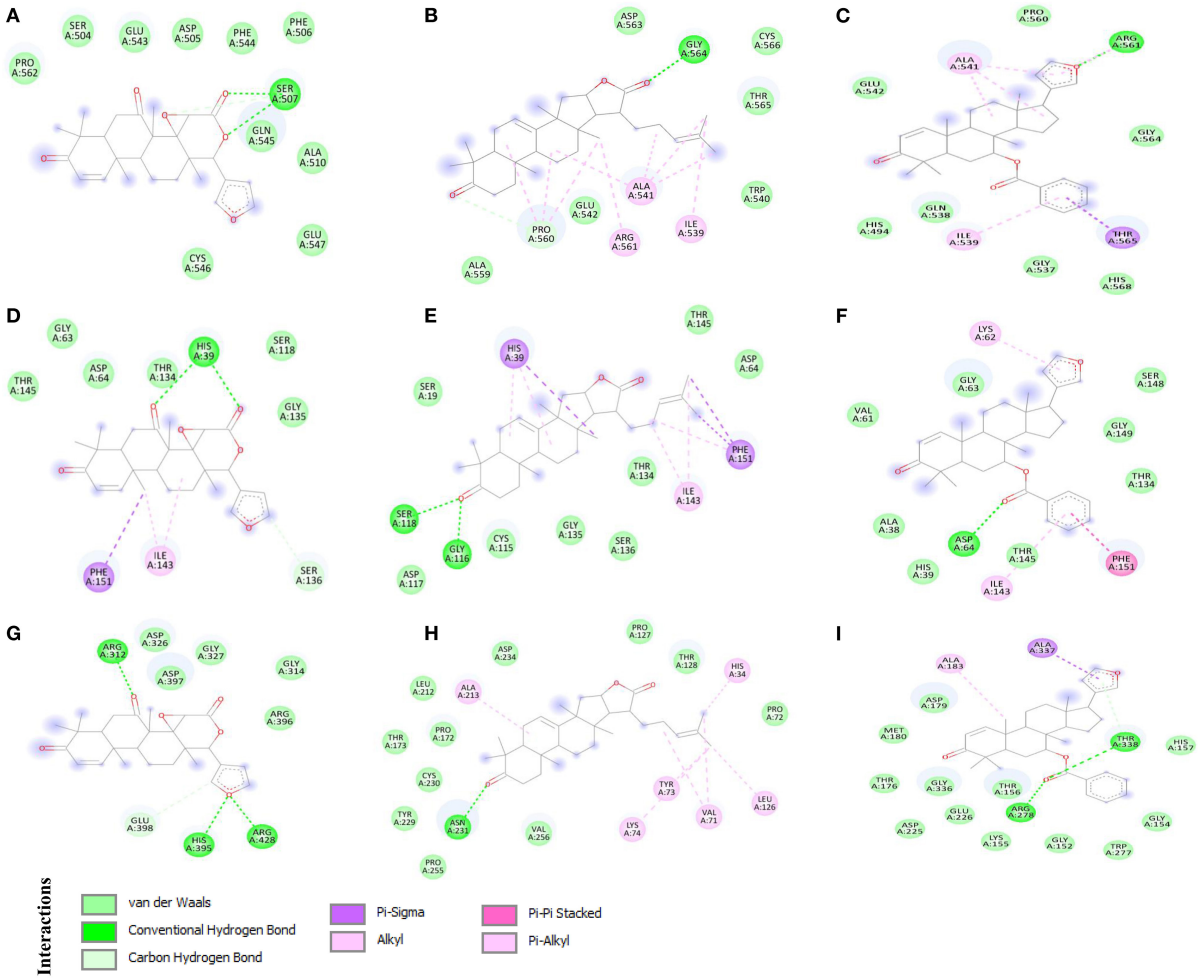
2D representation of the identified multi-target protein-ligand complexes and key amino acid residues contributing to the interactions. **(A)** CD163 scavenger receptor cysteine-rich domain 5 (CD163-SRCR5)-CID:1886, **(B)** CD163-SRCR5-CID:15560423, **(C)** CD163-SRCR5-CASID:104522-76-1, **(D)** Nsp4-CID:1886, **(E)** Nsp4-CID:15560423, **(F)** Nsp4-CASID:104522-76-1, **(G)** Nsp10-CID:1886, **(H)** Nsp10-CID:15560423, and **(I)** Nsp10-CASID:104522-76-1.

Discovery Studio Visualizer was used to analyse and visualize the protein-ligand interactions formed between phytochemicals and the target proteins. The multi-target compound 7-deacetyl-7-oxogedunin (CID:1886) formed two conventional hydrogen bonds (HBs) with CD163-SRCR5 and Nsp4, and three with Nsp10 (Figure 2). Moreover, it formed two conventional HBs at position Ser507. Besides, amino acid residues Ser504, Asp505, Phe506, Ala510, Glu543, Phe544, Gln545, Cys546, Glu547, and Pro562 were involved in van der Waals interactions (Figure 2A). 7-deacetyl-7-oxogedunin formed two conventional HBs with Nsp4 at position His39 and van der Waals interactions at Gly63, Asp64, Ser118, Thr134, Gly135, and Thr145. Further, Ser136 participated in interaction through a carbon hydrogen bond, and Ile143 and Phe151 formed alkyl and pi-sigma bonds, respectively (Figure 2D). Additionally, In case of Nsp10, it formed three HBs at positions Arg312, His395, and Arg428. Amino acid residues Gly314, Asp326, Gly327, Arg396, and Asp397 were involved in van der Waals interactions, and Glu398 formed a carbon hydrogen bond (Figure 2G).

Interestingly, kulactone (CID:15560423) interacted with CD163-SRCR5 and formed one conventional HB at position Gly564. The amino acid residues Trp540, Glu542, Ala559, Asp563, Thr565, and Cys566 contributed in interaction through van der Waals interactions. Additionally, Pro560 formed a carbon hydrogen bond, and Ile539, Ala541, and Arg561 formed alkyl bonds (Figure 2B). Kulactone also formed two conventional HBs with Nsp4 amino acid residues Gly116 and Ser118. The amino acid residues Ser19, Asp64, Cys115, Asp117, Thr134, Gly135, Ser136, and Thr145 formed van der Waals interactions. Additionally, kulactone formed pi-sigma and pi-alkyl bonds with His39, pi-alkyl bonds with Ile143, and pi-sigma and pi-alkyl bonds with Phe151 (Figure 2E). Kulactone also interacted and formed one conventional H-bond with Nsp10 at position Asn231. The amino acid residues Pro72, Pro127, Thr128, Pro172, Thr173, Leu212, Tyr229, Cys230, Asp234, Pro255, and Val256 contributed to protein-ligand interactions *via* van der Waals forces. In addition, kulactone formed alkyl and pi-alkyl bonds with amino acid residues His34, Val71, Tyr73, Lys74, Leu126, and Ala213 (Figure 2H).

Nimocin (CASID:104522-76-1) bonded with CD163-SRCR5 at position Arg561 by one conventional H-bond. In addition, the amino acid residues His494, Gly537, Gln538, Glu542, Pro560, Gly564, and His568 formed van der Waals interactions; Ile539, Ala541, and Arg561 formed alkyl bonds; and Thr565 formed a pi-sigma bond (Figure 2C). Nimocin also formed one conventional H-bond with Nsp4 at Asp64. Moreover, Ala38, His39, Val61, Gly63, Thr134, Thr145, Ser148, and Gly149 formed van der Waals interactions, and Lys62 and Ile143 formed pi-alkyl bonds. Additionally, one amino acid residue, Phe151, formed a pi-pi stacking interaction (Figure 2F). Nimocin interacted with Nsp10 amino acid residues Arg278 and Thr338 through conventional HBs. Other residues participating in the protein-ligand van der Waals interactions included Thr176, Asp179, Met180, Asp225, Glu226, Gly152, Gly154, Lys155, Thr156, His157, Trp277, and Gly336. Moreover, Ala183 and Ala337 formed pi-sigma and alkyl bonds with Nimocin, respectively (Figure 2I). The detailed information related to other phytochemicals is given in Table 1 and Supplementary Table 1.

### Drug-Likeness and Toxicity Assessment of the Multi-Target Phytochemicals

Prior to initiating experimental studies, newly discovered compounds must undergo predictive absorption, distribution, metabolism, excretion, and toxicity (ADMET) studies, which investigate the chemical nature in terms of pharmacological similarity. Therefore, ADMET analysis of the predicted multi-target phytochemicals was performed to assess their drug-likeness potential. A total of eight principal descriptors (molecular weight, LogP, H-bond donor and acceptor, topological polar surface area, mutagenicity, tumorigenicity, and irritation) were included in the study. The ADME related information (molecular weight, LogP, H-bond donor and acceptor, and topological polar surface area) were retrieved from IMPPAT and PubChem databases. Furthermore, phytochemical toxicity (T) was predicted by OSIRIS Property Explorer tool. Based on our analysis, the predicted multi-target compounds, 7-deacetyl-7-oxogedunin (CID:1886), kulactone (CID:15560423), and nimocin (CASID:104522-76-1) exhibited drug-like properties with no indication of mutagenicity, tumorigenicity, or irritation. Besides, their polar surface areas were <140 Å^2^, indicating high cell membrane permeability. The results of the drug-likeness analysis are shown in Table 2.

**Table 2:** Physicochemical properties and drug-likeness of identified multi-target phytochemicals.

**S.N**.	**Descriptors**	**7-Deacetyl-7-oxogedunin (CID:1886)**	**Kulactone (CID:15560423)**	**Nimocin (CASID:** **104522-76-1)**
1.	Molecular Weight (g/mol)	438.5	452.7	498.6
2.	LogP	4.20	7.06	7.53
3.	H-bond donor	0	0	0
4.	H-bond acceptor	6	3	4
5.	Topological Polar Surface Area (Å^2^)	86.1	43.4	56.51
6.	Mutagenic	No	No	No
7.	Tumorigenic	No	No	No
8.	Irritant	No	No	No

### Stability Analysis Through MD Simulation

MD simulation plays a remarkable role in confirming the stability of proteins and protein-ligand interactions. Therefore, a 100 ns MD simulation was conducted to examine the dynamic behavior and conformational stability of CD163-SRCR5, Nsp4, Nsp10, and their complexes with 7-deacetyl-7-oxogedunin (CID:1886), kulactone (CID:15560423), and nimocin (CASID:104522-76-1). The root mean square deviation (RMSD), root mean square fluctuation (RMSF), radius of gyration (Rg), number of HBs, and principal component analysis (PCA) were used to summarize the MD simulation results.

#### Conformational Stability Analysis

**Figure 3:**
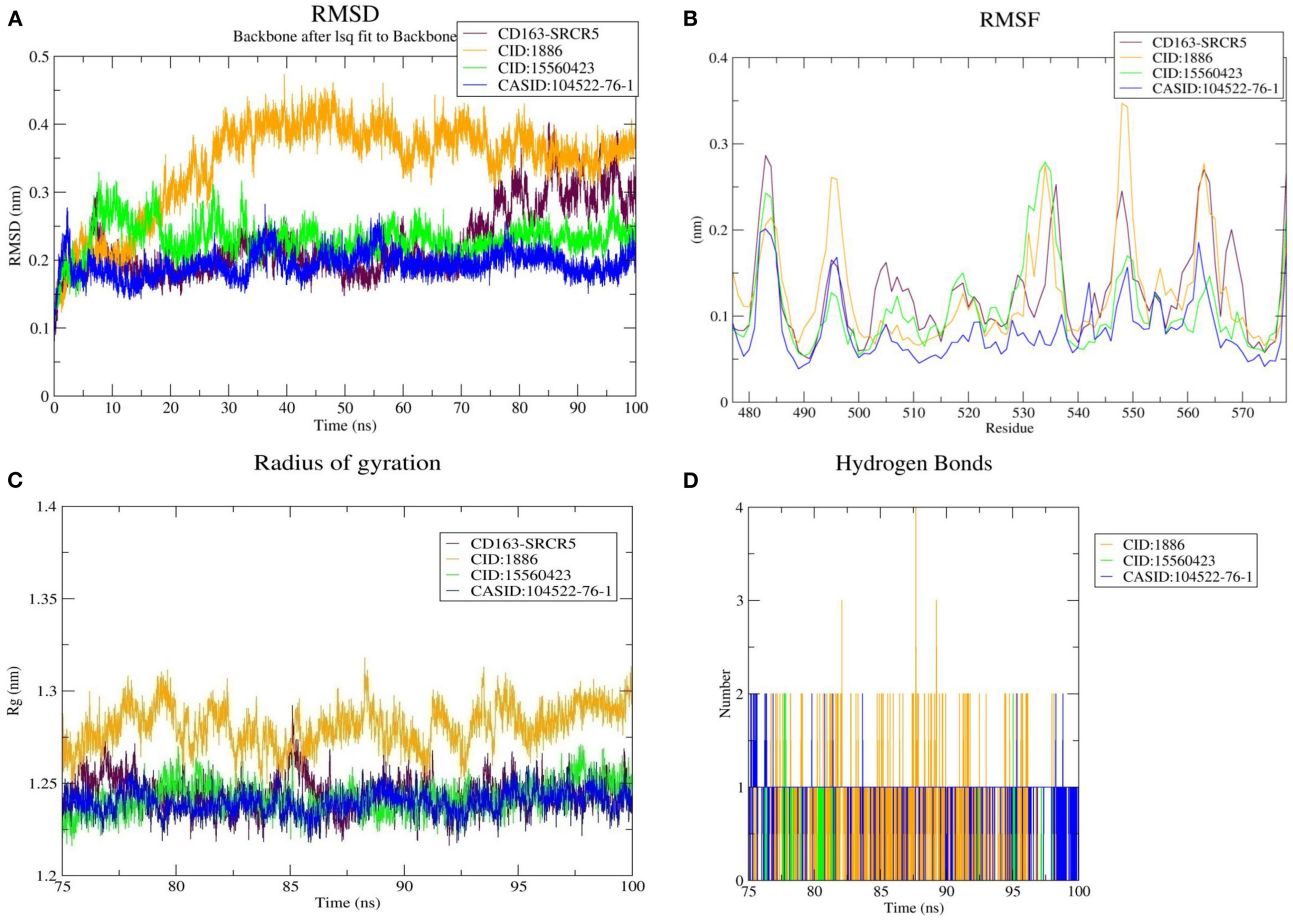
MD simulation results. **(A)** Root mean square deviation (RMSD) plot of CD163-SRCR5 and the CD163-SRCR5-phytochemical complexes, **(B)** root mean square fluctuation (RMSF), and **(C)** radius of gyration (Rg) plots of the CD163-SRCR5 and CD163-SRCR5-phytochemical complexes over the final 25 ns of the MD simulations. Maroon, orange, green, and blue colors represent CD163-SRCR5, CD163-SRCR5-CID:1886, CD163-SRCR5-CID:15560423, and CD163-SRCR5–CASID:104522-76-1, respectively. **(D)** A plot showing the number of hydrogen bonds formed over the final 25 ns trajectory for each respective complex. Orange, green, and blue colors represent CD163-SRCR5-CID:1886, CD163-SRCR5-CID:15560423, and CD163-SRCR5–CASID:104522-76-1, respectively.

We used RMSD to evaluate the conformational stability, an important parameter in measuring the protein stability with respect to their structure during MD simulation; In particular, the structure with smaller RMSD values is more stable than that with larger RMSD values. The backbone RMSD was plotted against time to assess conformational variations. The average RMSD of CD163-SRCR5 was calculated as 0.22 nm. Moreover, the RMSD values of CD163-SRCR5-CID:1886, CD163-SRCR5-CID:15560423, and CD163-SRCR5–CASID:104522-76-1 were 0.34, 0.23, and 0.19 nm, respectively (Figure 3A).

**Figure 4:**
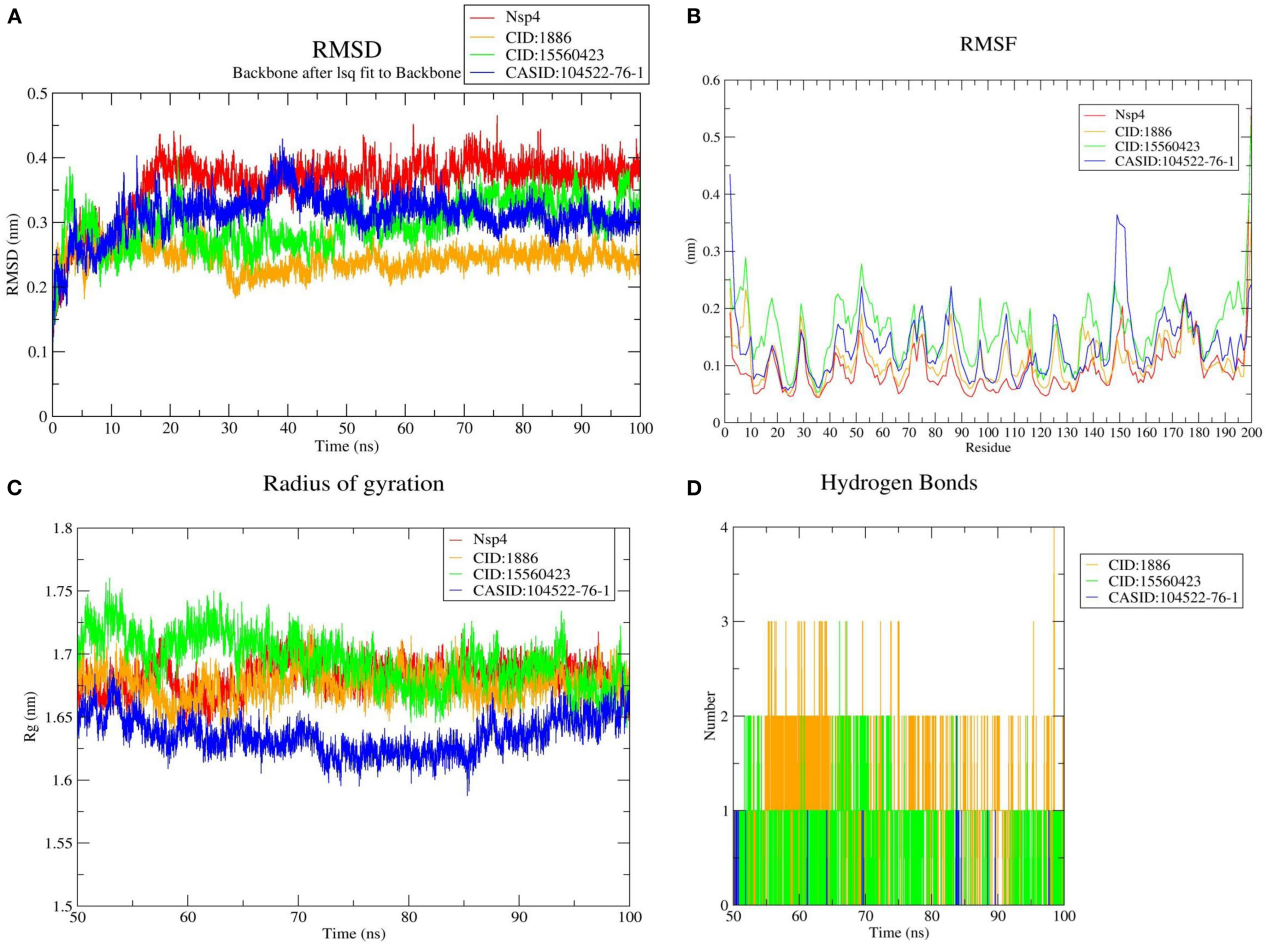
MD simulation results, **(A)** RMSD plot of Nsp4 and the Nsp4-phytochemical complexes, **(B)** RMSF, and **(C)** Rg plots of Nsp4 and the Nsp4-phytochemical complexes over the final 50 ns of the MD simulations. Red, orange, green, and blue colors represent Nsp4, Nsp4-CID:1886, Nsp4-CID:15560423, and Nsp4-CASID:104522-76-1, respectively. **(D)** A plot showing the number of hydrogen bonds formed over the final 50 ns trajectory for each respective complex. Orange, green, and blue colors represent Nsp4-CID:1886, Nsp4-CID:15560423, and Nsp4-CASID:104522-76-1, respectively.

**Figure 5:**
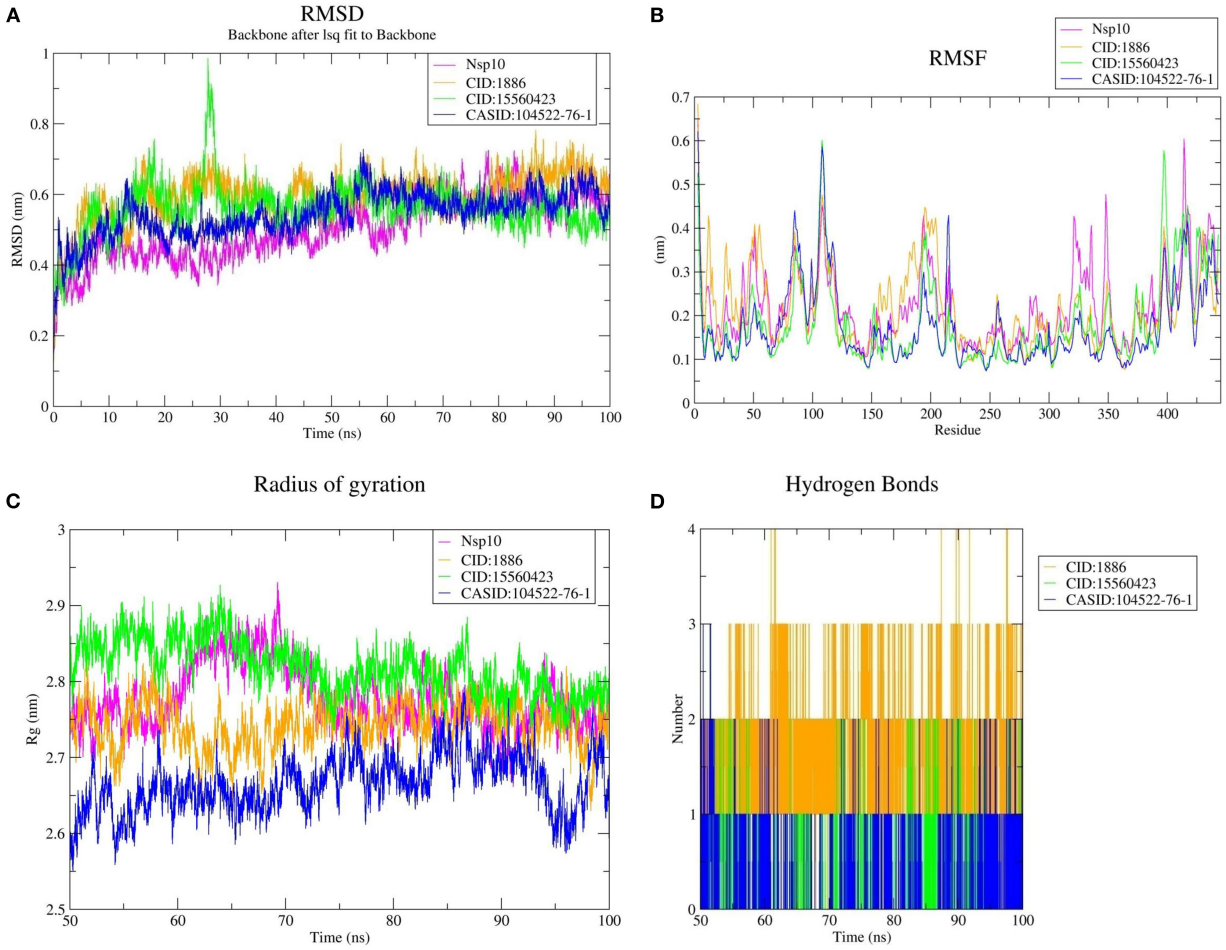
MD simulation results, **(A)** RMSD plot of Nsp10 and the Nsp10-phytochemical complexes, **(B)** RMSF, and **(C)** Rg plots of Nsp4 and the Nsp4-phytochemical complexes over the final 50 ns of the MD simulations. Magenta, orange, green, and blue colors represent Nsp10, Nsp10-CID:1886, Nsp10-CID:15560423, and Nsp10-CASID:104522-76-1, respectively. **(D)** A plot showing the number of hydrogen bonds formed over the final 50 ns trajectory for each respective complex. Orange, green, and blue colors represent Nsp10-CID:1886, Nsp10-CID:15560423, and Nsp10-CASID:104522-76-1, respectively.

Furthermore, the average RMSD value of Nsp4 was calculated as 0.35 nm, and the average RMSD values of its complexes Nsp4-CID:1886, Nsp4-CID:15560423, and Nsp4-CASID:104522-76-1, were 0.24, 0.29, and 0.31 nm, respectively (Figure 4A). Besides, the average RMSD values of Nsp10, Nsp10-CID:1886, Nsp10-CID:15560423, and Nsp10-CASID:104522-76-1 were calculated as 0.50, 0.59, 0.56, and 0.54 nm, respectively (Figure 5A). The RMSD graph shows that CD163-SRCR5, Nsp4 and Nsp10 as well as all the predicted hits reached equilibrium and produced a stable trajectory at 75 ns, 50 ns, and 50 ns, respectively. Therefore, the final 25 ns, 50 ns, and 50 ns trajectory for CD163-SRCR5, Nsp4 and Nsp10, respectively, were considered for the RMSF, Rg, number of HBs, and PCA.

#### Structural Flexibility Analysis

Protein structures are dynamic; they maintain their properties through structural flexibility, which can be measured by analyzing RMSF. Therefore, we analyzed the RMSF of native target proteins and their complexes with selected multi-target phytochemicals. The average RMSF value of CD163-SRCR5 was calculated as 0.12 nm. Additionally, the RMSF values of CD163-SRCR5-CID:1886, CD163-SRCR5-CID:15560423, and CD163-SRCR5–CASID:104522-76-1 were 0.12, 0.11, and 0.08 nm, respectively (Figure 3B). In case of Nsp4, the average RMSF value was 0.09 nm, and the average RMSF values of the complexes Nsp4-CID:1886, Nsp4-CID:15560423, and Nsp4-CASID:104522-76-1 were 0.10, 0.16, and 0.13 nm, respectively (Figure 4B). The average RMSF value of Nsp10, Nsp10-CID:1886, Nsp10-CID:15560423, and Nsp10-CASID:104522-76-1 were calculated as 0.22, 0.21, 0.18, and 0.17 nm, respectively (Figure 5B).

#### Structural Compactness Analysis

The structural compactness was measured by analyzing Rg values of the proteins and protein-ligand complexes. The time evolution of Rg values can be used to understand the mechanisms of protein structural compactness, stability, and folding. We calculated and plotted the Rg values of CD163-SRCR5, CD163-SRCR5-CID:1886, CD163-SRCR5-CID:15560423, and CD163-SRCR5–CASID:104522-76-1 systems from the MD trajectories. The average Rg values were 1.24, 1.28, 1.24, and 1.23 nm, respectively (Figure 3C). For Nsp4, Nsp4-CID:1886, Nsp4-CID:15560423, and Nsp4-CASID:104522-76-1, the average Rg values were 1.68, 1.67, 1.69, and 1.63 nm, respectively (Figure 4C). Besides, the average Rg values for Nsp10, Nsp10-CID:1886, Nsp10-CID:15560423, and Nsp10-CASID:104522-76-1 were calculated as 2.77, 2.74, 2.81, and 2.66 nm, respectively (Figure 5C).

#### Hydrogen Bond Analysis

HBs are significant because they bind the ligand with the target protein and regulate drug specificity and pharmacokinetics. Therefore, we measured the number of HBs formed during the interaction of the phytochemicals with CD163-SRCR5, Nsp4, and Nsp10. The number of HBs for the complexes CD163-SRCR5-CID:1886, CD163-SRCR5-CID:15560423, and CD163-SRCR5–CASID:104522-76-1 over the final 25 ns was 0–4, 0–2, and 0–2, respectively (Figure 3D). In case of complexes Nsp4-CID:1886, Nsp4-CID:15560423, and Nsp4-CASID:104522-76-1, the number of HBs over the final 50 ns was 0–4, 0–3, and 0–2, respectively (Figure 4D). Furthermore, Nsp10-CID:1886, Nsp10-CID:15560423, and Nsp10-CASID:104522-76-1 complexes showed 0–4, 0–2, and 0–3 HBs, respectively, over the final 50 ns of MD simulations (Figure 5D).

#### Principal Component Analysis

**Figure 6:**
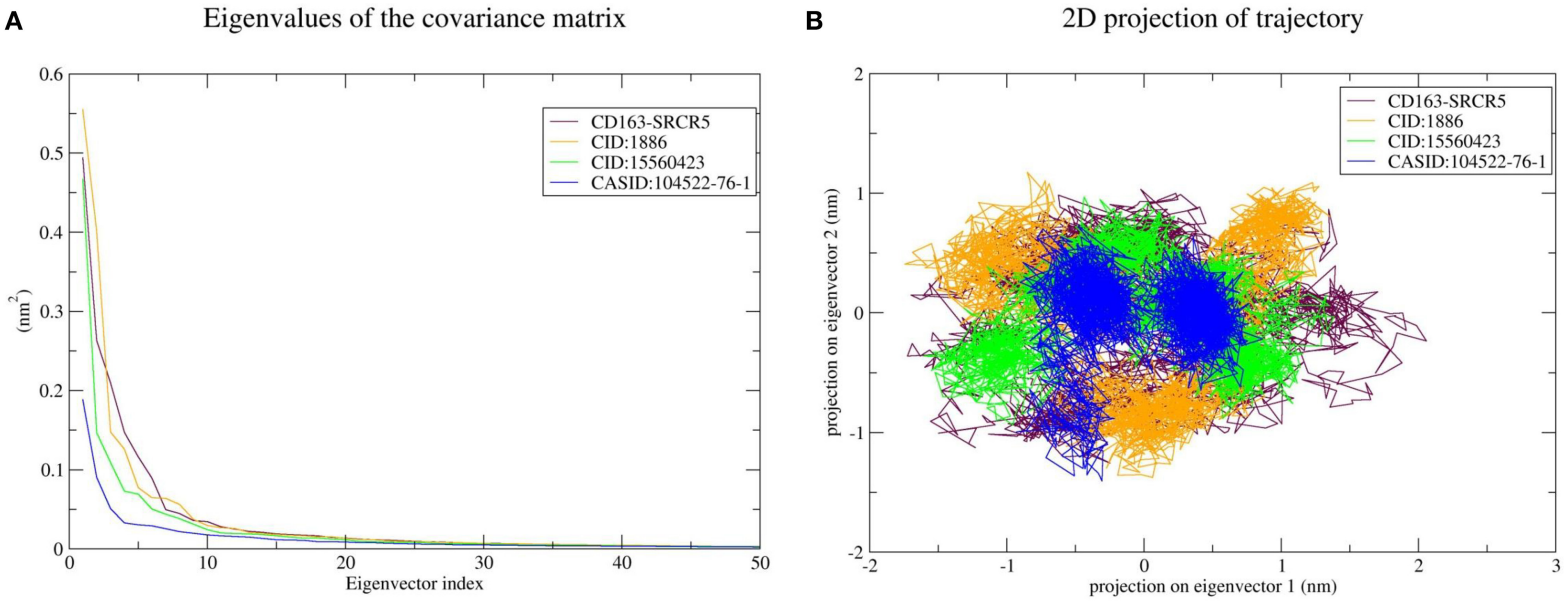
Principal component analysis of CD163-SRCR5 and the CD163-SRCR5-phytochemical complexes. **(A)** Plots of eigenvalues vs. the first 50 eigenvectors derived from the final 25 ns of MD simulations. **(B)** The first two eigenvectors representing the motion of CD163-SRCR5 and its complexes in phase space. Maroon, orange, green, and blue colors represent CD163-SRCR5, CD163-SRCR5-CID:1886, CD163-SRCR5-CID:15560423, and CD163-SRCR5–CASID:104522-76-1, respectively.

PCA was used to predict the significant motions that occur during ligand binding. The eigenvectors and eigenvalues were calculated using matrix diagonalization. The first 50 eigenvectors were considered to determine the changes in structural movement. The results revealed that out of fifty eigenvectors, the top 10 accounted for 79.56, 80.31, 77.20, and 66.66% of the motions for CD163-SRCR5, CD163-SRCR5-CID:1886, CD163-SRCR5-CID:15560423, and CD163-SRCR5–CASID:104522-76-1, respectively (Figure 6A).

**Figure 7:**
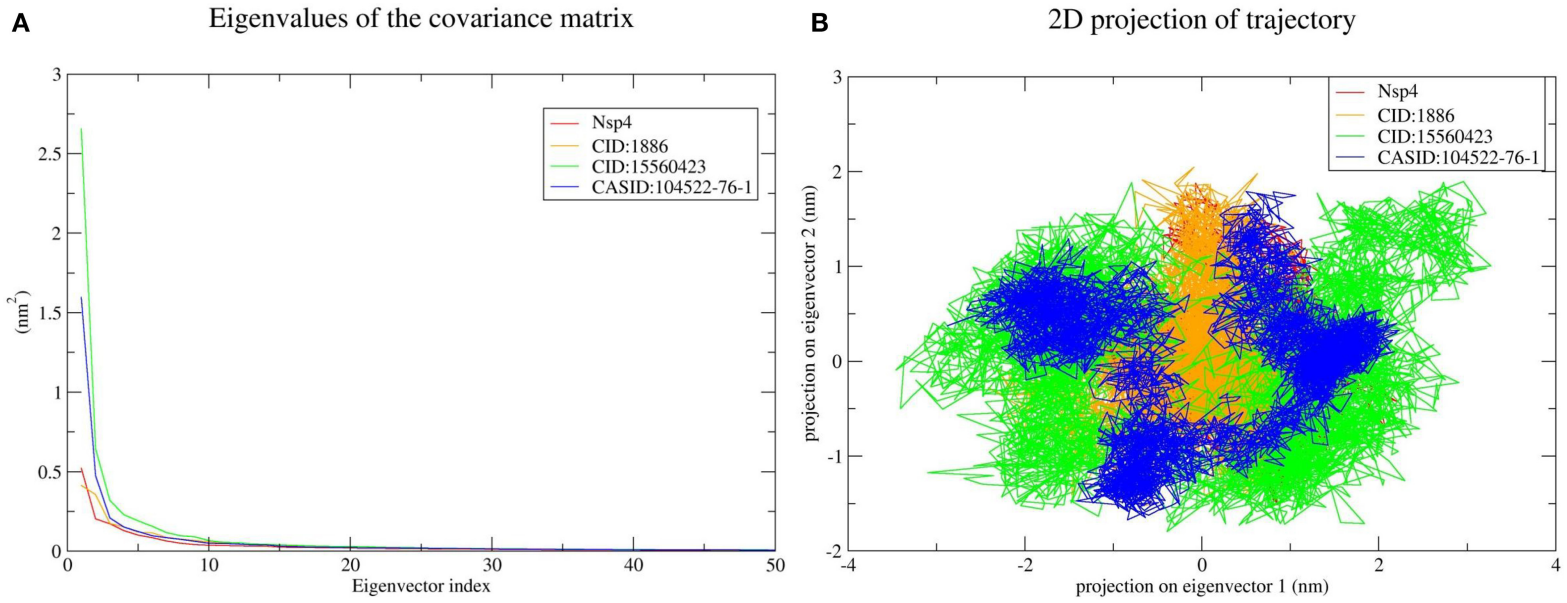
Principal component analysis of Nsp4 and the Nsp4-phytochemical complexes. **(A)** Plots of eigenvalues vs. the first 50 eigenvectors derived from the final 50 ns of MD simulations. **(B)** The first two eigenvectors representing the motion of Nsp4 and its complexes in phase space. Red, orange, green, and blue colors represent Nsp4, Nsp4-CID:1886, Nsp4-CID:15560423, and Nsp4-CASID:104522-76-1, respectively.

**Figure 8:**
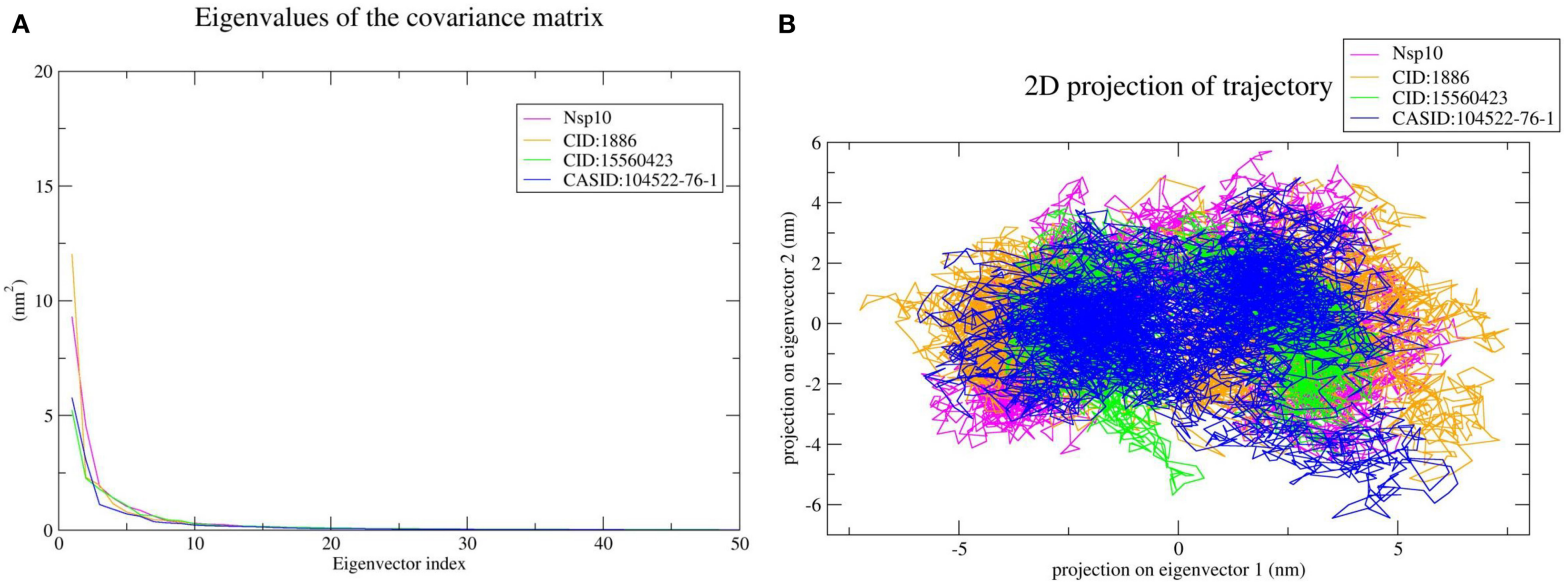
Principal component analysis of Nsp10 and Nsp10-phytochemical complexes. **(A)** Plots of eigenvalues vs. the first 50 eigenvectors derived from the final 50 ns of MD simulations. **(B)** The first two eigenvectors representing the motion of Nsp10 and its complexes in phase space. Magenta, orange, green, and blue colors represent Nsp10, Nsp10-CID:1886, Nsp10-CID:15560423, and Nsp10-CASID:104522-76-1, respectively.

The top 10 out of 50 eigenvectors accounted for 71.42, 70.30, 84.75, and 80.66% of the motions for Nsp4, Nsp4-CID:1886, Nsp4-CID:15560423, and Nsp4-CASID:104522-76-1, respectively (Figure 7A). Furthermore, 87.53, 89.30, 82.61, and 85.29% of motions were calculated for Nsp10, Nsp10-CID:1886, Nsp10-CID:15560423, and Nsp10-CASID:104522-76-1, respectively, using the top 10 selected eigenvectors (Figure 8A). Using PCA to generate 2D projection plots is another approach to analyse the dynamics of proteins and their complexes. Therefore, 2D plots for all the systems were generated from the first two eigenvectors to assess protein dynamics after ligand binding. CD163-SRCR5–CASID:104522-76-1 formed a more stable cluster than CD163-SRCR5, CD163-SRCR5-CID:1886, and CD163-SRCR5-CID:15560423 did (Figure 6B). Additionally, Nsp4-CID:1886 complex formed a more stable cluster than Nsp4, Nsp4-CID:15560423, and Nsp4-CASID:104522-76-1 did (Figure 7B). Furthermore, Nsp10-CID:15560423 complex formed a more stable cluster than Nsp10, Nsp10-CID:1886, and Nsp10-CASID:104522-76-1 did (Figure 8B).

### Validation of Phytochemical Affinities Toward Target Proteins Through MM-PBSA Studies

To validate the phytochemical affinities toward target proteins as predicted by MD simulations, the binding free energy of the simulated complex was estimated through MM-PBSA method. The last 5 ns of MD simulation trajectories were used to calculate binding free energies. The calculated binding free energy for CD163-SRCR5-CID:1886, CD163-SRCR5-CID:15560423, and CD163-SRCR5–CASID:104522-76-1 was −152.056, −81.761, and −75.324 kJ mol^−1^, respectively. The calculated binding free energy for Nsp4-CID:1886, Nsp4-CID:15560423, and Nsp4-CASID:104522-76-1 was −90.007, −81.437, and −94.841 kJ mol^−1^, respectively. Furthermore, the calculated binding free energy for Nsp10-CID:1886, Nsp10-CID:15560423, and Nsp10-CASID:104522-76-1 was −82.878, −88.943, and −108.489 kJ mol^−1^, respectively. The values of calculated van der Waals and electrostatic forces, polar solvation, SASA, and binding free energies are shown in Table 3.

**Table 3:** Affinities of multi-target neem phytochemical compounds with CD163-SRCR5, Nsp4, and Nsp10 (van der Waals and electrostatic forces, polar solvation, SASA, and binding free energy in kJ mol^−1^).

**Compound ID**	**Protein name**	**van der Waals energy**	**Electrostatic energy**	**Polar solvation energy**	**SASA energy**	**Binding energy**
CID:1886	CD163	−202.601 ± 13.426	−7.389 ± 8.306	74.783 ± 17.321	−16.850 ± 1.019	−152.056 ± 16.990
	Nsp4	−126.549 ± 10.052	−23.986 ± 7.917	71.794 ± 11.849	−11.266 ± 1.173	−90.007 ± 10.936
	Nsp10	−129.838 ± 9.336	−30.679 ± 12.409	89.749 ± 13.986	−12.110 ± 0.868	−82.878 ± 9.394
CID:15560423	CD163	−95.413 ± 10.557	−17.409 ± 5.646	40.499 ± 24.080	−9.437 ± 1.860	−81.761 ± 24.644
	Nsp4	−105.303 ± 9.772	−1.688 ± 5.886	36.785 ± 18.094	−11.230 ± 1.258	−81.437 ± 18.638
	Nsp10	−112.739 ± 10.567	−10.451 ± 9.192	45.978 ± 15.818	−11.732 ± 1.181	−88.943 ± 9.976
CASID:104522-76-1	CD163	−117.092 ± 10.784	−4.615 ± 4.952	58.741 ± 20.245	−12.359 ± 1.496	−75.324 ± 19.515
	Nsp4	−115.685 ± 12.455	−9.809 ± 3.295	42.480 ± 19.054	−11.827 ± 1.673	−94.841 ± 19.088
	Nsp10	−162.469 ± 12.424	−24.887 ± 8.799	95.972 ± 13.774	−17.105 ± 1.537	−108.489 ± 11.464

## Discussion

The swine industry suffers enormous economic losses as a result of PRRSV infection (49). Current vaccines do not provide complete protection and the virus develops rapidly with new strains appearing frequently (50). Antiviral therapy may be an important practice for preventing PRRSV infection (49). For generations, the neem plant has been widely utilized in traditional medicine (51). According to previous research, neem contains chemicals that have potent antiviral properties. The inhibitory potential of neem extracts against poliovirus, HSV, influenza, HIV, and coxsackie B group virus has been well-documented. Similarly, it is effective in inhibiting dengue virus type 2 and other viruses during their replication step (51).

In multiple studies over the years, computational methods have been proven effective in discovering novel natural compounds capable of efficiently binding to molecular targets, such as proteins. The interactions between natural compounds and target proteins are analyzed for the purpose of drug discovery (52, 53). These interactions can also be utilized in the investigation of antiviral compounds. Furthermore, an additional advantage of these strategies is safety due to natural or plant-based origin of the compounds (54). Our findings support this strategy and indicate that the selected compounds have a potential to act as antiviral lead compounds against PRRSV.

The study screened and analyzed 70 neem phytochemicals targeting the porcine CD163-SRCR5 (32, 33), PRRSV Nsp4 (35), and Nsp10 (36). The porcine CD163-SRCR5 is a key virus entry mediator, and gene-edited pigs resistant to PRRSV can be generated through CRISPR/Cas9 technology (55). However, these pigs are prohibited in most countries. Therefore, CD163-SRCR5 is one of the promising molecular drug targets. Besides, the role of other selected targets, Nsp4 and Nsp10, in virus replication and disease development are well-illustrated. Therefore, targeting host and pathogen proteins with antiviral multi-target natural compounds might be an efficient way to combat PRRSV.

The free energy change associated with a binding process is known as binding affinity. The ligand binding affinity measures the strength of the binding interaction with the target protein and is directly linked to ligand potency. As a result, its assessment is critical in the domains of drug discovery and personalized medicine (56). Furthermore, the free energy is negative in favorable reactions. Therefore, ligand-protein binding is improved by lowering the binding energy, and low binding energy corresponds with high binding affinity of protein-ligand complexes. Molecular docking was used for phytochemical screening to predict their binding energy toward target proteins. The three most promising compounds, 7-deacetyl-7-oxogedunin (CID:1886), kulactone (CID:15560423), and nimocin (CASID:104522-76-1) were chosen as multi-target ligands based on their lowest binding energies. Besides, they showed strong affinities toward target proteins in terms of different interactions with key amino acid residues. The results of physicochemical property and toxicity prediction analyses suggested that the selected multi-target compounds act as drugs and could be considered for further evaluation (40). In particular, 100 ns MD simulation was conducted to evaluate the dynamic behavior of the systems, i.e., macromolecular target and its docked complexes. This is a popular method for estimating macromolecule conformational dynamics before and after ligand interaction, and the simulated data may be used to calculate the binding free energy of small molecules over time (57). During RMSD analysis, CD163-SRCR5–CASID:104522-76-1 complex was the most stable as compared to other CD163-SRCR5 complexes; however, the other complexes were stabilized after 75 ns. Additionally, Nsp4-CID:1886 complex was the most stable as compared to other Nsp4 complexes, and the other complexes were stabilized after 50 ns. Finally, Nsp10-CASID:104522-76-1 was the most stable compared to other Nsp10 complexes; the other complexes were stabilized after 50 ns. Based on overall RMSD results, we concluded that all the complexes stabilized during the simulation time. To assess the amino acid residue mobility and fluctuation, we conducted RMSF analysis, during which we found that the protein-ligand interaction changes the protein structure geometry. It is worth noting that a correct conformation is essential for all proteins to perform their native functions (58, 59). We observed that CD163-SRCR5–CASID:104522-76-1, NSP4-CID:1886, and NSP10-CASID:104522-76-1 showed less fluctuation than other complexes. Further, Rg analysis was conducted to determine the compactness of proteins and its complexes during MD simulation. The folding and unfolding of target proteins upon small molecule binding can be investigated through Rg analysis (59). It is well-known that the high Rg values correspond with less compactness; therefore, we concluded that the CASID:104522-76-1 complexes with CD163-SRCR5, Nsp4, and Nsp10 were more compact than other protein-ligand complexes included in the study. However, all the complexes in the simulations reached a stable peak after 75 ns (CD163-SRCR5) and 50 ns (Nsp4 and Nsp10). Therefore, all predicted complexes were compact and stable during protein-ligand interaction analysis.

The most essential directional interaction in biological macromolecules is hydrogen bonding, responsible for protein structural stability and selectivity in protein-ligand interactions (60). It plays a vital role in the establishment of molecular interactions between proteins and ligands. We calculated the number of HBs vs. time for all the complexes. Based on our analysis, we concluded that each selected compound stably interacted with the target protein binding cavity and provided a stable complex. In addition, PCA was conducted to analyse essential dynamics, i.e., correlated motions in the target proteins before and after ligand binding (59). The difference in CD163-SRCR5, Nsp4, and Nsp10 motions was observed after ligand binding. This difference implied that ligand binding causes structural and motional changes in the protein. 2D projection plots were also generated to further analyse the first two eigenvectors and predict phase space dynamics of the target proteins and their protein-ligand complexes. As a result, we concluded that these three compounds can be used as multi-target lead compounds for PRRSV inhibition.

Furthermore, the binding affinities of predicted multi-target phytochemicals with CD163-SRCR5, Nsp4, and Nsp10 were validated by MM-PBSA binding energy calculations. This is a popular method for predicting binding free energy since it is more accurate than most scoring functions used in MD and is commonly employed in biomolecular research, including protein-ligand interactions (61–65). The results of MM-PBSA calculations showed that the predicted multi-target phytochemicals had strong affinity with target proteins. Finally, we concluded that these protein-ligand complexes were energetically stable and could act as novel natural inhibitors against PRRSV.

## Conclusion

PRRSV causes serious illnesses in pigs, including reproductive impairment or failure and respiratory disease. It is prevalent in many countries throughout the world, resulting in huge financial losses to the swine industry. To date, no effective antiviral compounds targeting the multiple proteins responsible for its pathogenesis have been identified. Therefore, this study aimed to identify effective neem compounds that inhibit the multiple proteins responsible for disease development. The present work has utilized vetinformatics approaches, including molecular docking, pharmacokinetics, toxicity assessment, and MD simulation, followed by MM-PBSA binding free energy calculations, all of which have suggested three compounds as potential multi-target drug candidates. Namely, 7-deacetyl-7-oxogedunin (CID:1886), kulactone (CID:15560423), and nimocin (CASID:104522-76-1) inhibited the activity of CD163-SRCR5, Nsp4, and Nsp10. Additionally, the three identified compounds can be used individually or in combination against the virus. However, further *in vitro* and *in vivo* research is needed to establish the antiviral and multi-target inhibitory potential of these compounds against the PRRSV.

## Data Availability Statement

The original contributions presented in the study are included in the article/Supplementary Material, further inquiries can be directed to the corresponding author/s.

## Author Contributions

J-MK designed the experiments and supervised the research. RKP performed experiments, analyzed results, and wrote the manuscript. D-YK and BL helped in analysis and provided valuable inputs. All authors read and approved the final manuscript.

## Funding

This research was supported by the Basic Science Research Program through the National Research Foundation of Korea (NRF) funded by the Ministry of Education (NRF-2018R1A6A1A03025159).

## Conflict of Interest

The authors declare that the research was conducted in the absence of any commercial or financial relationships that could be construed as a potential conflict of interest.

## Publisher's Note

All claims expressed in this article are solely those of the authors and do not necessarily represent those of their affiliated organizations, or those of the publisher, the editors and the reviewers. Any product that may be evaluated in this article, or claim that may be made by its manufacturer, is not guaranteed or endorsed by the publisher.
